# Meta-Analysis of the Association between Tea Intake and the Risk of Cognitive Disorders

**DOI:** 10.1371/journal.pone.0165861

**Published:** 2016-11-08

**Authors:** Qing-Ping Ma, Chen Huang, Qiao-Yun Cui, Ding-Jun Yang, Kang Sun, Xuan Chen, Xing-Hui Li

**Affiliations:** Tea Research Institute, College of Horticulture, Nanjing Agricultural University, Nanjing, Jiangsu Province, China; Banner Alzheimer's Institute, UNITED STATES

## Abstract

**Background:**

Alzheimer’s disease is a common neurodegenerative disorder in elderly. This study was aimed to systematically evaluate the association between tea intake and the risk of cognitive disorders by meta-analysis.

**Methods and Findings:**

PubMed, Embase and Wanfang databases were systematically searched and a total of 26 observational studies were included in this study. Odds ratios (ORs) and the corresponding 95% confidence intervals (CIs) were calculated and pooled by using fixed or random effects models according to the degree of heterogeneity.

**Results:**

The overall pooled analysis indicated that tea intake could significantly reduce the risk of cognitive disorders (OR = 0.65, 95%CI = 0.58–0.73). Subgroup analyses were conducted based on study design, population, frequency of tea drinking and type of cognitive disorders. The results showed that tea drinking was significantly associated with the reduced incidence of cognitive disorders in all of subgroups based on study design and frequency of tea drinking. In particular, tea drinking was inversely associated with the risk of cognitive impairment (CoI), mild cognitive impairment (MCI), cognitive decline and ungrouped cognitive disorders. Moreover, for population subgroups, the significant association was only found in Chinese people.

**Conclusion:**

Our study suggests that daily tea drinking is associated with decreased risk of CoI, MCI and cognitive decline in the elderly. However, the association between tea intake and Alzheimer’s disease remains elusive.

## Introduction

Cognitive disorders are a category of mental disease that affects memory, language, learning and problem solving. Alzheimer’s disease (AD) and cognitive impairment (CoI) are two common cognitive disorders in elderly and negatively affect the elders’ life. Cognitive disorders are caused by a complex of genetics and environmental factors [1,2]. Because of limited treatment of cognitive disorders, the prevention or onset delay of the disease through modification of risk factors such as lifestyles are proposed [3,4]. Some lifestyles, such as folic acid supplementation [5], flavonoid-rich food [6] and caffeine contained drinks [7] have been reported to be inversely associated with the risk of cognitive disorders.

Tea, a flavonoid-rich and caffeine contained drink, is popular worldwide. Recent studies proposed that drinking tea may reduce the risk of AD and CoI [8]. Kuriyama et al. [9] stated that the higher green tea consumption was associated with the lower prevalence of CoI in humans. Ide et al. [10] also found that the green tea intake could improve the cognitive function or delay the progression of cognitive dysfunction in elders. However, some other researchers presented the opposite results showing no obvious association between tea drinking and cognitive disorders [11,12].

Several systematic review or meta-analyses on this issue have been reported. However, these analyses only examined the effect of one major component in tea such as caffeine [13] or flavonoids [14], and some other caffeine contained food or drinks like coffee were also considered in these analyses.

In this study, we performed a new meta-analysis to specifically and systematically evaluate the association between tea drinking and the incidence of cognitive disorders such as AD and CoI by using pooled analysis of the published observational studies. Since China has the most tea drinkers in the world, the related Chinese reports were included in our meta-analysis.

## Materials and Methods

### Literature search

We searched PubMed (http://www.ncbi.nlm.nih.gov/pubmed/), Embase (https://www.embase.com/) and Chinese Wanfang database (http://www.wanfangdata.com.cn/) up to September, 2015 using the search terms of “tea AND (Alzheimer disease OR dementia OR cognitive*)” in English and Chinese. We also searched the references from the included studies and relevant reviews to identify additional publications. The study selection process was performed following the PRISMA (Preferred Reporting Items for Systematic Review and Meta-Analyses) statement [15]. The PRISMA checklist for this meta-analysis was shown in S1 File.

### Selection criteria

We selected the studies which conformed with the following criteria: 1) only observational studies with case-control, cohort and cross-sectional design were considered due to the lack of relevant double-blind placebo controlled trials; 2) the study reported the relationship between tea consumption and cognitive disorders such as AD, cognitive decline and CoI in elderly; 3) the study provided the data for calculating the estimates of odds ratio (OR) and the corresponding 95% confidence interval (CI).

The animal studies, reviews, reports and studies with unavailable data were excluded. If two or more studies shared the same population, we only selected the latest one. Two of us (Ma and Huang) completed the literature search process independently and the disagreements were resolved by discussion.

### Data extraction and quality assessment

Huang and Cui independently extracted the main information of the included studies using the predesigned form and assessed the methodological quality of the case-control and cohort studies using the Newcastle-Ottawa Scale (NOS) [16]. The NOS contained 3 aspects with 8 items: selection of the participants (4 items), comparability of the cohorts/cases and controls (1 item) and the exposure/outcomes (3 items). The total score was 9 stars with 1 star for each item and 2 stars for the item of comparability. In this study, 7, 4–6 and < 4 stars were considered as high, moderate and low quality, respectively. The discrepancies were discussed with the third reviewer (Yang).

### Statistical analysis

We pooled the odds ratio (OR) and the corresponding 95% confidence interval (CI) using fixed or random effects models according to the degree of heterogeneity. The heterogeneity was assessed by *I*^*2*^ statistic, which measures the extent of true heterogeneity dividing the difference between the result of the *Q* test and its degrees of freedom (*k*– 1) by the *Q* value itself, and multiplied by 100 [17]. *I*^*2*^ > 50% was considered as heterogeneity and the pooled analysis was conducted using a random effects model. Otherwise, the fixed effect model was used. We conducted the subgroup analysis according to study design, population, drink frequency and type of cognitive disorder. For drink frequency, the studies without description of the drink frequency were classified into “ungrouped subgroup”. These analyses were conducted using Stata 13 software. In addition, publication bias was assessed using Begg’s and Egger’s test.

## Results

### Study selection

**Fig 1:**
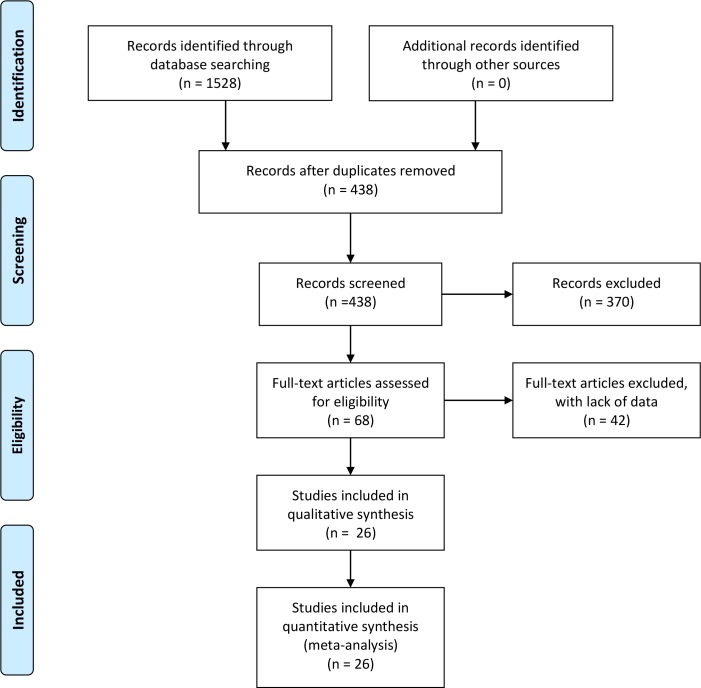
Study selection process for this meta-analysis.

The study selection process was shown in Fig 1. Our initial search resulted in retrieval of 1 528 articles (397 from PubMed, 1 027 from Embase and 104 from Wanfang). Firstly, we took out 1 090 duplicate records by preliminary screening. Secondly, 370 animal studies or reviews which obviously deviated from inclusion criteria were removed by reading titles and abstracts. Thirdly, we removed 42 articles after reviewing the full-texts and the reasons for exclusion were listed in S2 File. Finally, 26 studies were included in this meta-analysis [6,9,11,12,18–39].

### Study characteristics

Table 1 summarized the characteristics of the 26 selected studies including 10 case-control studies, 4 cohorts and 12 cross-sectional studies. These studies contained 52 503 participants distributed in Asia, Europe, Australia and America. All of the participants were 50 years and older. For diagnosis of the study outcomes, Diagnostic and Statistical Manual of Mental Disorders-IV (DSM-IV) was used for dementia diagnosis, National Institute of Neurological and Communicative Diseases and Stroke/Alzheimer's Disease and Related Disorders Association (NINCDS-ADRDA) for AD diagnosis and Mini-Mental State Examination (MMSE) for CoI diagnosis. Cognitive impairment contains all the patients falling in between healthy and demented states in these included studies. Mild cognitive impairment (MCI) was defined as an early state of cognitive impairment in the included studies. In addition, cognitive decline was defined as a drop of ≥1–2 MMSE scores from the baseline. Some other auxiliary criteria such as Montreal Cognitive Assessment (MoCA), Mini-Cog and Petersen’s criteria were also used in combination for diagnosis of these cognitive disorders (Table 1).

**Table 1:** Characteristics of the included studies in this meta-analysis.

Study	Population	Study design	n (male/female)	mean consumption	Assessment of cognitive status	Cognitive results	Adjust factors
Broe 1990 [32]	Australian (52–96)	hospital based case-control	340 (170/170)	drinking vs. never; >4cups/d vs. < 4cups/d	Neurology of Aging Schedule, MMSE, comprehent neuropsychological assessment, NINCDS-ADRDA for probable or possible AD	drinking in cases 162, drinking in controls 166; >4cups/d in cases: 73, in control 58; never drink in cases 8, in controls 4	Age, sex and, where possible, the general practice of origin.
Chen 2012 [18]	Chinese (≥65)	prospective nested case-control study	5,691 (1,389/4,302)	tea drinking vs. not drinking	MMSE-r less than 18 for cognitive decline	OR = 0.82 (0.68, 1.00)	NA
Cheng 2014 [19]	Chinese (>60)	cross-sectional	3,885 (2,379/1,506)	tea drinking vs. not drinking	DSM-IV, and clinical evaluation for dementia; HDS and CMS for CoI	484 CI patients in 1927 non tea drinkers, 437 CI patients in 1958 tea drinkers	NA
Dai 2006 [35]	Japanese Americans in King County, Washington (≥65)	cohort with mean 6.4 y follow up	1589 (725/864)	1–2 times/wk, 3 or more times/wk vs. less often than weekly	NINCDS-ADRDA for AD	1–2 times per week HR = 1.49 (0.43–5.16); 3 times or more per week 1.70 (0.67–4.33); Less Often Than Weekly 1.00	Years of education, gender, regular physical activity, body mass index, baseline CASI score, olfaction diagnostic group, total energy intake, intake of saturated, monounsaturated, and polyunsaturated fatty acids, ApoE genotype, smoking status, alcohol drinking, supplementation of vitamin C, vitamin E, and multivitamin, and tea drinking, and fruit and vegetable juice drinking, dietary intake of vitamin C, vitamin E, and *Beta*-carotene
Ding 2012 [20]	Chinese (>60)	case-control	3,141 (1,438/1,703)	≥3 d/wk vs. not drinking	C-MMSE and AVLT for CoI	OR = 0.36 (0.17, 0.75) for AD	Sex, age, education, marriage, BMI, ApoE 4, family economic status in childhood, experience significant adverse events, smoking, drinking, doing physical job before retire, have physical training habit, have many sisters and brothers, family history of dementia, history of hypertension, diabetes, coronary heart disease, stroke and hyperlipidemia
						OR = 0.74 (0.56, 0.98) for MCI	
Eskelinen 2009 [38]	Finland (50.4 ±6.0 for women and 71.3±4.0 for men)	cross-sectional	1,409 (543/875)	≥1 cup/day vs. not drinking	MMSE≤24 and DSM-IV for dementia; MMSE≤24 and NINCDS-ADRDA for AD	OR = 1.04 (0.59, 1.84) for dementia, OR = 0.91 (0.48, 1.71) for AD, and OR = 1.27 (0.84, 1.91) for all the demented).	Midlife smoking, SBP, serum total cholesterol, BMI, and physical activity.
Forster 1995 [11]	English (≥65 with mean onset age of 55.9±3.9)	case-control	218	>4 cups/d of tea vs. not drinking	NINCDS-ADRDA criteria for AD, DSM-III-R criteria for dementia, MMSE for CoI	OR = 1.40 (0.81, 1.63)	NA
Guo 2011 [21]	Chinese (≥65)	hospital based case-control	214 (105/109)	tea drinking vs. not drinking	AD diagnosis: C-MMSE, MoCA <24; CDR >1; HIS≤4; FAQ≥5; and NINCDS-ADRDA	controls: 93 tea drinkers (17 <4times/wk and 76 >4times/wk) and 58 never drink; AD cases: 33 tea drinkers (15 <4 times/wk and 18 >4 times/wk) and 30 never drink	NA
Huang 2009 [48]	Chinese (90–108)	cross-sectional	681 (223 /458)	drinking former vs. not drinking	MMSE<24 for CoI	men: OR = 0.917 (0.344, 2.449); women: OR = 0.862 (0.265, 0.907)	Age, sex, sleep habits, educational levels, religion habits, and temperament.
Kuriyama 2006 [9]	Japanese (≥70)	cross-sectional	1,003	3 cups/wk vs. 4–6 cups/wk or 1 cup/d, and 2 cups/d (100 mL/cup)	MMSE ≤26 for CoI	For green tea consumption, the OR = 1.00 (reference) for<3 cups/wk, 0.62 (0.33,1.19) for 4–6 cups/wk or 1 cup/d, and 0.46 (0.30, 0.72) for 2 cups/d. Corresponding ORs were 1.00 (reference), 0.60 (0.35, 1.02), and 0.87 (0.55, 1.38) for black or oolong tea	NA
Lian 2013 [22]	Chinese (≥60)	case-control	240 (104/136)	drinking everyday vs. not drinking	C-MMSE and DSM-IV for MCI	OR = 0.73 (0.47, 1.13)	NA
Lindsay 2002 [12]	Canadian (≥65)	cohort with 5y follow up	4,088 (1,718/2,370)	tea drinking vs. not drinking	mMMSE <78/100 and clinical evaluation for AD	OR = 1.12(0.78, 1.61)	Age, sex, and education.
Luo 2015 [23]	Chinese (≥65)	case-control	1,981 (817/1,168)	tea drinking vs. not drinking	Petersen’s criteria for MCI	102 MCI patients in 932 tea drinkers and 197 patients in 1049 non-drinkers	NA
Ng 2008 [24]	Chinese living in Singapore (≥55)	cross-sectional	2,194	drinking tea with low, medium and high levels vs. not drinking	MMSE ≤23 as CoI, a drop in MMSE score of ≥1 point as cognitive decline	For CoI: Low intake 0.56 (0.40,0.78), Medium 0.45 (0.27, 0.72), high 0.37 (0.14, 0.98); for cognitive decline: Low intake 0.74 (0.54, 1.00), Medium 0.78 (0.55, 1.11), High 0.57 (0.32, 1.03)	Age, sex, education, smoking, alcohol consumption, BMI (continuous), hypertension, diabetes, heart disease, stroke, depression, APOE 4, physical activities, social and productive activities, vegetable and fruit consumption, fish consumption, and coffee consumption.
Noguchi-Shinohara 2014 [25]	Japanese (≥60)	cohort with mean follow up of 4.9y	490	For green tea, drinking moderate and every day vs. not drinking; for black tea, drinking 1–7 d/wk vs. not drinking	MMSE <24 for CoI	For dementia, the OR were 0.90 (0.34, 2.35) for 1–6 days/week and 0.26 (0.06, 1.06) for every day. For cognitive decline (MCI or dementia), the OR were 0.47 (0.25, 0.86) and 0.32 (0.16, 0.64) for 1–6 days/week and every day, respectively.	Age and sex, history of hypertension, diabetes mellitus, hyperlipidemia, education, ApoE E4 carrier status, alcohol drinking, smoking, physical activities and/or hobbies, and coffee and black tea consumption.
Nurk 2009 [6]	Norwegian (70–74)	cross-sectional	2,031 (914/1,117)	tea drinking vs. not drinking	mMMSE ≤10 for CoI	OR = 0.33 (0.16, 0.69)	All values are adjusted for sex, education, vitamin supplement use (multivitamins, folate, and vitamins B, C, D, or E), smoking status,history of CVD, diabetes, and total energy intake.
Pan 2012 [26]	Chinese (>60)	cross-sectional	897 (434/463)	drinking occasionally, drinking often vs. not drinking	MoCA and MMSE for MCI	OR = 0.751 (0.593, 0.951)	Age, education, sleep, social activity and study
Shen 2015 [31]	Chinese (≥60)	cross-sectional	9,375 (4,548/4,827)	<2 cups/d, 2-4cups/d and ≥4 cups/d vs. not drinking (250 mL/cup)	C-MMSE for CoI	compared with non-consumption participants, those who consumed < 2 cups/d, 2–4 cups/d, and ≥4 cups/d were observed ORs of 0.77 (0.56, 1.07), 0.62 (0.47, 0.81), and 0.49 (0.36, 0.66), respectively.	Age, sex, race, education, marriage, tea concentration, tea categories, physical examinations (BMI, WHR, SBP, DBP), family status (family income, have children or not) and disease situation (history of present illness and family history of hypertension, diabetes, CHD, AD, PD), behavioral risk factors (cigarette smoking, alcohol consumption, and physical activities), dietary intake (vegetables, fruits, red meat, fish, beans, milk), nutrition supplement, depression and ADL
Song 2007 [27]	Chinese (>60)	cross-sectional	3,047 (2,618/429)	tea drinking vs. not drinking	CCMD2-R, DSM-IV and ICD-10 for dementia; MoCA and MMSE for MCI	371 MCI patients in 1788 tea drinkers and 350 patients in 1259 non-drinkers	NA
Sun 2012 [33]	Chinese (≥60)	case-control	168 (48/120)		C-MMSE for CoI, clinical test, DSM- IV, ADL and CSDD for dementia	OR 0.778 (0.607, 0.996)	Hypertension, smoking, drinking, physical activity, live alone, insomnia, bland diet, high cholesterol, high blood glucose, uric acid, thin and fat
Wang 2012 [28]	Chinese (>60)	case-control	174	drinking everyday, 1–4 d/wk, occasionally vs. not drinking	DSM-IV, NINCDS-ADRDA and clinical test for VD	drinking occasionally OR = 0.52 (0.15, 1.82), 1-4d/wk OR = 0.41 (0.11, 1.57), everyday OR = 0.35 (0.18, 0.68)	Economic income, hypertension, education and location
Wang 2014 [36]	Chinese (≥65)	cohort with 2 year follow up	223 (70/153)	drink sometimes, often vs. never drinking	A drop of ≥ 2 MMSE points as cognitive decline	146 non cognitive decline: 30 never drink green tea, 19 drink sometimes and 97 drink often; 74 cognitive decline: 26 never drink green tea, 8 drink sometimes and 40 drink often	Age, non-Chinese speaking background and education, and a formal diagnosis of dementia
Wu 2011 [37]	Chinese (≥65)	cross sectional	2,119 (1017/1102)	< 1 time/wk, > 1 time/wk vs. not drinking	MMSE<24 for CoI	less than once per week 1.14 (0.82–1.59) more than once per week 0.99 (0.75–1.3)	Age, gender, education level, marital status, social support, hyperlipidemia, stroke, physical function, depressive symptoms, self-rated health, cigarette smoking, leisure-time, physical activity, fruits and vegetables consumption, coffee intake, multivitamin intake, BMI
Xu 2012 [29]	Chinese (≥50)	cross-sectional	3,485 (1,126/2,359)	green tea drinking vs. not drinking	C-MMSE, CDT and Mini-Cog for CoI	OR = 0.56 (0.40, 0.79)	NA
Yao 2010 [39]	Chinese (≥60)	cross-sectional	2,809 (1,010/1,799)	tea drinking daily vs. not drinking	C-MMSE	64 CoI patients in 1244 tea drinkers; 131 CoI patients in 1503 non-tea drinkers	NA
Yin 2012 [30]	Chinese (≥65)	cross-sectional	1,011 (410/601)	tea drinking vs. not drinking	Petersen’s criteria for MCI diagnosis	44 MCI patients in 687 tea drinkers and 23 patients in 324 non-drinkers	NA

Notes: MMSE: Mini-Mental State Examination

C-MMSE (Chinese revised MMSE): ≤24 for people with more than 6 years education, ≤20 for people with 1–6 years of education, ≤17 for illiteracy mild cognitive impairment

HDS: Hasegawa Dementia Scale, HIS: Hachinski ischemia score, MoCA: montreal cognitive assessment scale, ADL: activities of daily living scale

CSDD: the Chinese version of the Cornell scale for depression in dementia, AVLT: Auditory verbal learning test, CMS: Clinical Memory Scale

Table 2 showed the quality assessment of the included case-control studies and cohorts. The studies were awarded relatively high quality.

**Table 2:** The quality assessment of the included studies.

Study	Selection	Comparability	Exposure/Outcome	Total
Case-control studies				
Broe 1990	☆☆☆☆	☆	☆☆☆	8
Chen 2012	☆☆☆☆	☆	☆	6
Ding	☆☆☆	☆	☆☆	6
Forster 1995	☆☆☆☆	☆	☆	6
Guo 2011	☆☆☆☆	☆☆	☆☆	8
Lian 2013	☆☆☆☆	☆☆	☆☆	8
Lindsay 2002	☆☆☆	☆☆	☆☆	7
Luo 2015	☆☆☆☆	☆	☆☆	7
Wang 2012	☆☆☆☆	☆☆	☆☆	8
Sun 2012	☆☆☆☆	☆☆	☆	7
Cohort studies				
Dai 2006	☆☆☆	☆☆	☆☆	7
Ng 2008	☆☆☆	☆☆	☆	6
Noguchi-Shinohara 2014	☆☆☆☆	☆☆	☆☆	8
Wang 2014	☆☆☆☆	☆	☆☆	7

### Association of tea intake and the risk of cognitive disorders

**Fig 2:**
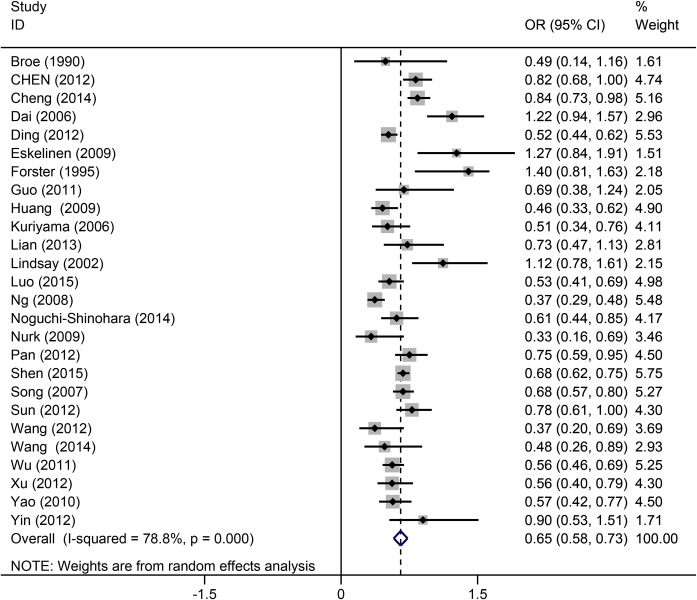
Overall pooled analysis of association between tea intake and the cognitive disorders.

As shown in Fig 2, a significant heterogeneity (*I*^*2*^ = 78.8%) was found. Thus, a random effects model was used in this meta-analysis. The forest plot showed that tea drinking was inversely associated with the risk of cognitive disorders (OR = 0.65, 95%CI = 0.58–0.73).

### Subgroup analyses

**Fig 3:**
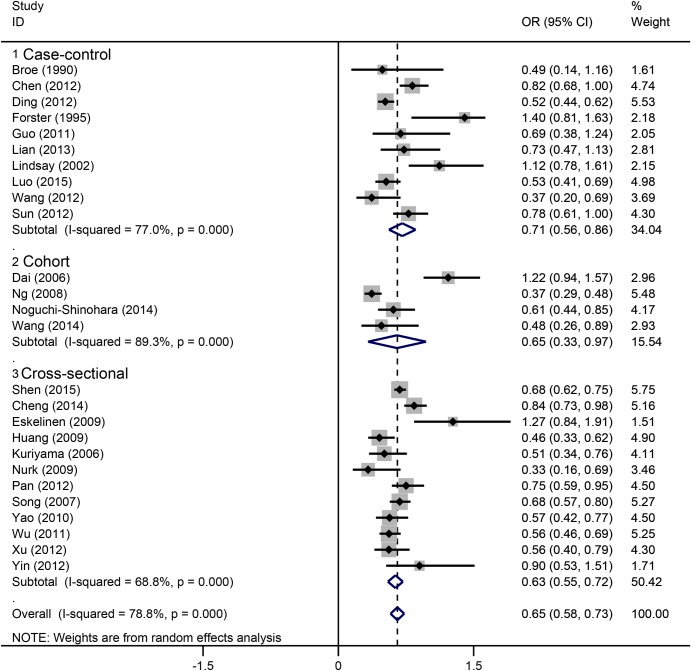
Subgroup analysis of association between tea intake and the cognitive disorders based on study design.

**Fig 4:**
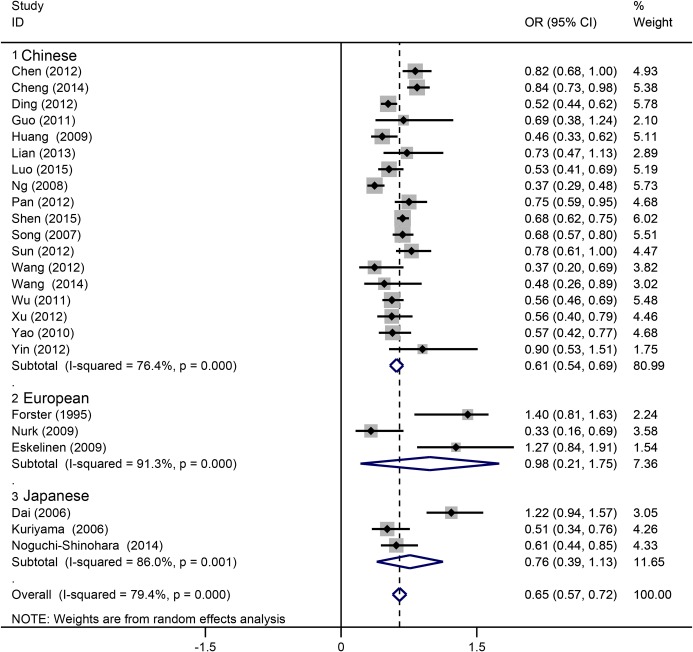
Subgroup analysis of association between tea intake and the cognitive disorders based on population.

**Fig 5:**
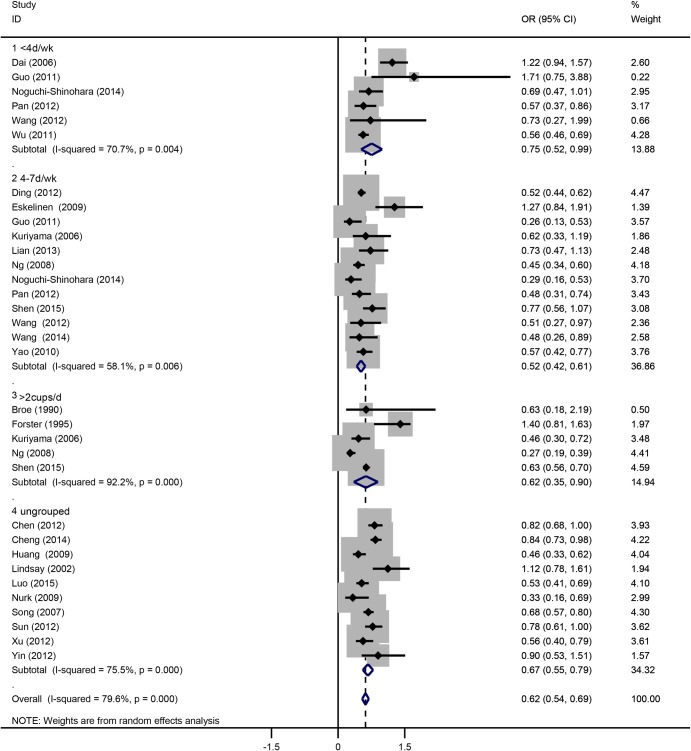
Subgroup analysis of association between tea intake and the cognitive disorders based on tea drinking frequency. Ungrouped means studies without information on drinking frequency.

**Fig 6:**
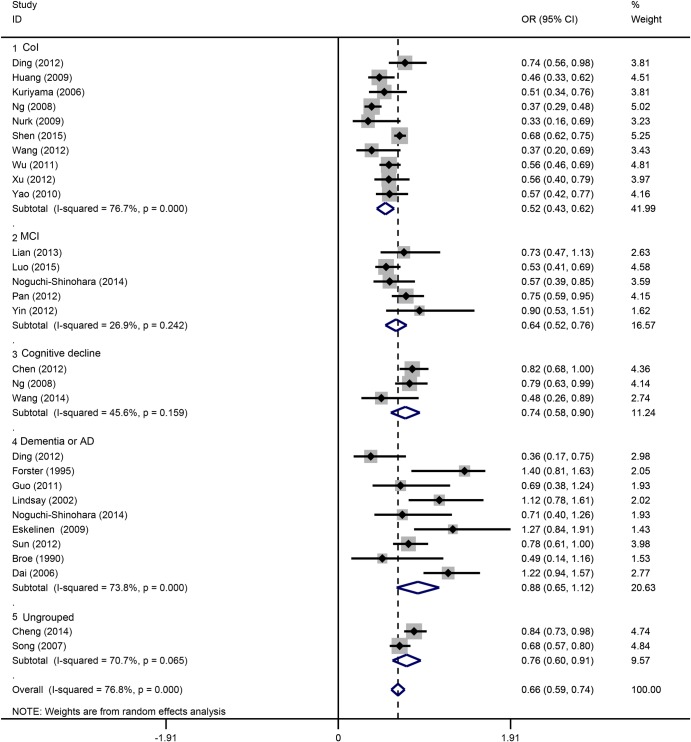
Subgroup analysis of association between tea intake and the cognitive disorders based on type of cognitive disorders.

Based on the study design, we classified all studies into three subgroups: case-control, cohort and cross-sectional (Fig 3). Each of the three subgroups showed an inverse correlation between tea drinking and cognitive disorder. In population subgroups (Fig 4), tea drinking could significantly reduce the risk of cognitive disorders in Chinese (OR = 0.61, 95%CI = 0.54–0.69). However, no significant associations were found in European (OR = 0.98, 95%CI = 0.21–1.75) and Japanese populations (OR = 0.76, 95%CI = 0.39–1.13). In subgroups by drinking frequency (Fig 5), all of the tea drinkers showed significant lower risk of cognitive disorders compared to those not drinking or rare drinking. In subgroup analysis based on type of cognitive disorders (Fig 6), drinking tea could significantly lower the risk of CoI (OR = 0.52, 95%CI = 0.43–0.62), MCI (OR = 0.64, 95%CI = 0.52–0.76), cognitive decline (OR = 0.74, 95%CI = 0.58–0.90) and unclassified cognitive disorder (OR = 0.76, 95%CI = 0.60–0.91). However, no significant association was found between tea intake and dementia or AD (OR = 0.88, 95%CI = 0.65–1.12).

### Publication bias

**Fig 7:**
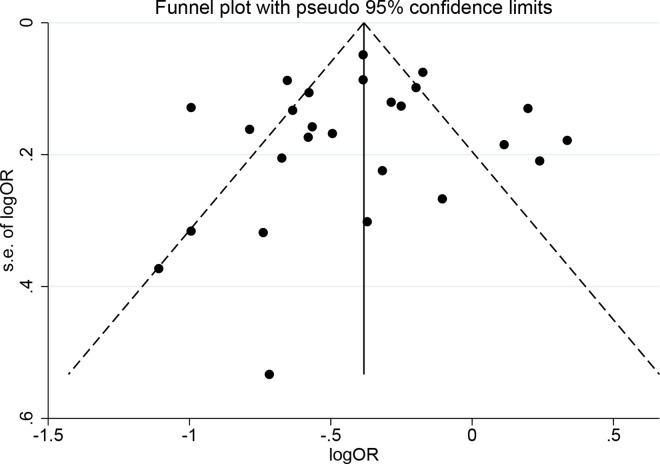
Funnel plot for assessment of publication bias.

Publication bias was assessed according to the overall pooled analysis. Begg’s test (*P* = 0.691), Egger’s test (*P* = 0.707) and the symmetrical funnel plot (Fig 7) indicated no publication bias in this meta-analysis.

## Discussion

In the current meta-analysis, we found that tea intake could significantly reduce the risk of cognitive disorders in elderly. Subgroup analyses showed similar results in subgroups. Our results were the opposite of a recent meta-analysis which found no association between caffeine from coffee or tea and cognitive disorders [13]. Caffeine is an important component in tea and coffee. Many experimental studies also showed the benefits of caffeine on cognitive function. For an instance, in aged mice with AD, caffeine could reverse CoI and decrease brain amyloid-β levels [40]. But unlike coffee, there are so many other beneficial elements aside from caffeine in tea including the polyphenols which have antioxidant effect [41,42]. Accumulating evidence showed the neuroprotective activity of the main catechin (-)-epigallocatechin-3-gallate (EGCG) from tea [43]. Chronic green tea EGCG could improve learning and memory deficits in diabetic rats via retardation of oxidative stress and modulation of nitric oxide [44]. In Alzheimer transgenic mice, EGCG showed the protective effect through modulating cleavage of amyloid precursor protein and reducing cerebral amyloidosis [45]. As above, the cognition protective effect of tea may be owing to the catechin and caffeine components.

Tea has several subtypes based on the processing technology, such as green, black and oolong tea. These subtypes of tea have different content of catechins and caffeine. Among the tea types commonly consumed, the highest catechins and caffeine were found in green tea and black tea, respectively [46]. In the present meta-analysis, an included study [24] showed significant decrease of the CoI incidence in black and oolong tea drinkers (OR = 0.55, 95%CI = 0.40–0.76) and green tea drinkers (OR = 0.42, 95%CI = 0.25–0.69). Because the black and oolong tea were combined in one category, we were not sure if black tea has a different effect from green tea on preventing the progression of CoI. In another study [29], green tea drinking was also associated with reduced risk of CoI (OR = 0.56, 95%CI = 0.40–0.79). However, whether green tea is more beneficial cannot be certain due to lack of comparison with other types of tea. Therefore, tea types should be considered in future studies.

In subgroup analysis, we found that tea intake could significantly reduce the risk of cognitive disorders in Chinese, but not in Japanese and Europeans. Because only one study each considered Australian [32] and Canadian patients [12], they were not included in subgroup analysis. Both two studies showed no significant association between tea intake and cognitive disorders (Fig 2). We think that the major reason is the different outcomes among the included studies. For the Chinese group, the major outcome was CoI or MCI. However, for European, Australian and Canadian groups, the major outcome was AD. This is consistent with the subgroup analysis in terms of outcomes which showed no association between tea intake and AD. A Japanese study [25] showed an obvious association between tea intake and MCI, but no obvious association was found in the outcome of AD (Fig 6). Nonetheless, we cannot draw a conclusion that there has no association between tea drinking and AD, because of lack of included studies, especially lack of non-Chinese studies. As well, the larger sample sizes in CoI studies than AD studies would influence the pooled result (Table 1). In addition to the above reasons, the origin of the studies, the locations/regions of studied populations and the sample size would also affect the results of the pooled analyses. Except for tea drink frequency, the intake duration is also an important factor influencing the overall pooled result. However, only one study [21] considered the tea intake duration, and drinking tea more than 10 years did not decrease the risk of AD compared to the people drinking less than 10 years (OR = 0.43, 95%CI = 0.17–1.05). This result should be confirmed by larger sample studies in future. It also reminds the researchers considering the effect of tea intake duration in further experiment design.

Our meta-analysis has strengths, such as no publication bias. Publication bias is an important factor influencing the quality and reliability of meta-analysis. In this meta-analysis, symmetric distribution of the included studies would improve the reliability of the statistical analysis to an extent. Subgroup analyses could evaluate the effect of the study design, population, drinking frequency and different cognitive disorders on the overall pooled result. In addition, our meta-analysis included more Chinese studies than the previous one [13]. Tea drinkers may be easier to find in Chinese people.

Nevertheless, this meta-analysis has several limitations. Firstly, there were no double-blind placebo controlled trials regarding this topic. All of the included studies were observational studies. Also, some confounders were not adjusted in the original studies. Therefore, the confounders would cause bias in our pooled results. Secondly, it is difficult to ensure the tea drink frequency and volume of the participants. Some included studies did not classify the tea drinkers by drink frequency and volume. The cognitive protective effects of tea are usually dose dependent. If the tea drink history of the participants was unclear, the pooled analysis would be neutralized. Thirdly, different diagnostic criteria were used in the original studies. Finally, significant heterogeneity was found in our meta-analysis. One of the major causes might be the various cut-off value of MMSE (range from 10 to 26) for CoI diagnosis. Besides, female and older population may have a higher risk of cognitive disorders [47]. Thus, some other potential confounders like age, gender and lifestyles may partially contribute to the heterogeneity.

In conclusion, we meta-analyzed 26 observational studies and found that daily tea drinking could decrease the risk of CoI, MCI and cognitive decline in elderly. However, no association was found between tea intake and AD. Further studies are needed to confirm our findings.

## Supporting Information

S1 File The PRISMA Checklist. (DOC)

S2 File The reasons for exclusion of the 42 full-text reviewed studies. (PDF)
