# Supplementary material for: Meta-Analysis of the Association between Tea Intake and the Risk of Cognitive Disorders
Source: PLoS One. 2016 Nov 8;11(11):e0165861. doi: 10.1371/journal.pone.0165861 (PMC5100989; doi:10.1371/journal.pone.0165861)
Supplement: S2 File — (PDF) [file pone.0165861.s002.pdf]

Reasons for full-text excluded studies

A total of 42 studies were excluded after reviewing full-texts. Of these, 20 studies were excluded, because the outcomes were not cognitive disorders considered in this meta-analysis [1-20]. Another 11 studies were excluded due to no available estimates or the data for calculating the estimates [21-31]. Additional 5 studies were excluded by reason of unevaluated effect of tea with the influence of other food like coffee or vegetables [32-36]. Moreover, 3 investigations which only considered the consumptions of some components in tea [37-39]. Finally, 2 reviews [40,41] and 1 animal experiment report [42] were excluded.

References

1. Butchart C, Kyle J, McNeill G, Corley J, Gow AJ, et al. (2011) Flavonoid intake in relation to cognitive function in later life in the Lothian Birth Cohort 1936. Br J Nutr 106: 141-148.
2. De Bruin EA, Rowson MJ, Van Buren L, Rycroft JA, Owen GN (2011) Black tea improves attention and self-reported alertness. Appetite 56: 235-240.
3. Dodd FL, Kennedy DO, Riby LM, Haskell-Ramsay CF (2015) A double-blind, placebo-controlled study evaluating the effects of caffeine and L-theanine both alone and in combination on cerebral blood flow, cognition and mood. Psychopharmacology (Berl) 232: 2563-2576.
4. Durlach PJ (1998) The effects of a low dose of caffeine on cognitive performance. Psychopharmacology (Berl) 140: 116-119.
5. Einother SJ, Martens VE, Rycroft JA, De Bruin EA (2010) L-theanine and caffeine improve task switching but not intersensory attention or subjective alertness. Appetite 54: 406-409.
6. Foxe JJ, Morie KP, Laud PJ, Rowson MJ, de Bruin EA, et al. (2012) Assessing the effects of caffeine and theanine on the maintenance of vigilance during a sustained attention task. Neuropharmacology 62: 2320-2327.
7. Giesbrecht T, Rycroft JA, Rowson MJ, De Bruin EA (2010) The combination of L-theanine and caffeine improves cognitive performance and increases subjective alertness. Nutr Neurosci 13: 283-290.
8. Haskell CF, Kennedy DO, Milne AL, Wesnes KA, Scholey AB (2008) The effects of L-theanine, caffeine and their combination on cognition and mood. Biol Psychol 77: 113-122.
9. Henning SM, Fajardo-Lira C, Lee HW, Youssefian AA, Go VL, et al. (2003) Catechin content of 18 teas and a green tea extract supplement correlates with the antioxidant capacity. Nutr

Cancer 45: 226-235.

10. Hindmarch I, Quinlan PT, Moore KL, Parkin C (1998) The effects of black tea and other beverages on aspects of cognition and psychomotor performance. Psychopharmacology (Berl) 139: 230-238.
11. Kelly SP, Gomez-Ramirez M, Montesi JL, Foxe JJ (2008) L-theanine and caffeine in combination affect human cognition as evidenced by oscillatory alpha-band activity and attention task performance. J Nutr 138: 1572s-1577s.
12. Pan C-W, Wang X, Ma Q, Sun H-P, Xu Y, et al. (2015) Cognitive dysfunction and health-related quality of life among older Chinese. Sci Rep 5.
13. Rogers PJ, Smith JE, Heatherley SV, Pleydell-Pearce CW (2007) Time for tea: mood, blood pressure and cognitive performance effects of caffeine and theanine administered alone and together. Psychopharmacology 195: 569-577.
14. Schmidt A, Hammann F, Wolnerhanssen B, Meyer-Gerspach AC, Drewe J, et al. (2014) Green tea extract enhances parieto-frontal connectivity during working memory processing. Psychopharmacology (Berl) 231: 3879-3888.
15. Scholey A, Downey LA, Ciorciari J, Pipingas A, Nolidin K, et al. (2012) Acute neurocognitive effects of epigallocatechin gallate (EGCG). Appetite 58: 767-770.
16. Seeram NP, Henning SM, Niu Y, Lee R, Scheuller HS, et al. (2006) Catechin and caffeine content of green tea dietary supplements and correlation with antioxidant capacity. J Agr Food Chem 54: 1599-1603.
17. Smit HJ, Rogers PJ (2000) Effects of low doses of caffeine on cognitive performance, mood and thirst in low and higher caffeine consumers. Psychopharmacology (Berl) 152: 167-173.
18. Smith AP (2009) Caffeine, cognitive failures and health in a non-working community sample. Hum Psychopharmacol 24: 29-34.
19. Tomata Y, Kakizaki M, Nakaya N, Tsuboya T, Sone T, et al. (2012) Green tea consumption and the risk of incident functional disability in elderly Japanese: the Ohsaki Cohort 2006 Study. Am J Clin Nutr 95: 732-739.
20. Cumming RG, Klineberg RJ (1994) A study of the reproducibility of long-term recall in the elderly. Epidemiology 5: 116-119.
21. Arab L, Biggs ML, O'Meara ES, Longstreth WT, Crane PK, et al. (2011) Gender differences in tea, coffee, and cognitive decline in the elderly: the Cardiovascular Health Study. J Alzheimers Dis 27: 553-566.
22. Corley J, Jia X, Kyle JA, Gow AJ, Brett CE, et al. (2010) Caffeine consumption and cognitive function at age 70: the Lothian Birth Cohort 1936 study. Psychosom Med 72: 206-214.
23. Feng L, Li J, Ng TP, Lee TS, Kua EH, et al. (2012) Tea drinking and cognitive function in oldest-old Chinese. J Nutr Health Aging 16: 754-758.
24. Hameleers PA, Van Boxtel MP, Hogervorst E, Riedel WJ, Houx PJ, et al. (2000) Habitual caffeine consumption and its relation to memory, attention, planning capacity and psychomotor performance across multiple age groups. Hum Psychopharmacol 15: 573-581.
25. Park SK, Jung IC, Lee WK, Lee YS, Park HK, et al. (2011) A combination of green tea extract and l-theanine improves memory and attention in subjects with mild cognitive impairment: a double-blind placebo-controlled study. J Med Food 14: 334-343.
26. Ramsden CM, Kinsella GJ, Ong B, Storey E (2008) Performance of everyday actions in mild Alzheimer's disease. Neuropsychology 22: 17-26.

27. Vercambre MN, Berr C, Ritchie K, Kang JH (2013) Caffeine and cognitive decline in elderly women at high vascular risk. J Alzheimers Dis 35: 413-421.
28. Wightman EL, Haskell CF, Forster JS, Veasey RC, Kennedy DO (2012) Epigallocatechin gallate, cerebral blood flow parameters, cognitive performance and mood in healthy humans: a double-blind, placebo-controlled, crossover investigation. Hum Psychopharmacol 27: 177-186.
29. Xing M, Ai YM, He RL, Gao JW, Song PP, et al. (2012) Current status and influencing factors regarding quality of life among patients with Alzheimer's disease. Zhonghua Liu Xing Bing Xue Za Zhi 33: 606-609.
30. Feng L, Gwee X, Kua EH, Ng TP (2010) Cognitive function and tea consumption in community dwelling older Chinese in Singapore. J Nutr Health Aging 14: 433-438.
31. Zeng Y, Chen H, Ni T, Ruan R, Feng L, et al. (2015) GxE interactions between FOXO genotypes and drinking tea are significantly associated with prevention of cognitive decline in advanced age in China. J Gerontol A Biol Sci Med Sci 70: 426-433.
32. Chin AV, Robinson DJ, O'Connell H, Hamilton F, Bruce I, et al. (2008) Vascular biomarkers of cognitive performance in a community-based elderly population: the Dublin Healthy Ageing study. Age Ageing 37: 559-564.
33. Commenges D, Scotet V, Renaud S, Jacqmin-Gadda H, Barberger-Gateau P, et al. (2000) Intake of flavonoids and risk of dementia. Eur J Epidemiol 16: 357-363.
34. Del Felice A, Broggio E, Valbusa V, Gambina G, Arcaro C, et al. (2014) Transient epileptic amnesia mistaken for mild cognitive impairment? A high-density EEG study. Epilepsy Behav 36: 41-46.
35. Drzezga A, Lautenschlager N, Siebner H, Riemenschneider M, Willoch F, et al. (2003) Cerebral metabolic changes accompanying conversion of mild cognitive impairment into Alzheimer's disease: a PET follow-up study. Eur J Nucl Med Mol Imaging 30: 1104-1113.
36. Shimada H, Makizako H, Doi T, Yoshida D, Tsutsumimoto K, et al. (2013) Combined prevalence of frailty and mild cognitive impairment in a population of elderly Japanese people. J Am Med Direc Association 14: 518-524.
37. Khokhar S, Magnusdottir SGM (2002) Total Phenol, Catechin, and Caffeine Contents of Teas Commonly Consumed in the United Kingdom. J Agr Food Chem 50: 565-570.
38. Liang W, Binns C, Lee AH, Huang R, Hu D (2008) The reliability of dietary and lifestyle information obtained from spouses in an elderly chinese population. Asia Pac J Public Health 20: 87-93.
39. Yahya HM, Day A, Lawton C, Myrissa K, Croden F, et al. (2015) Dietary intake of 20 polyphenol subclasses in a cohort of UK women. Eur J Nutr.
40. Eskelinen MH, Kivipelto M (2010) Caffeine as a protective factor in dementia and Alzheimer's disease. J Alzheimers Dis 20 Suppl 1: S167-174.
41. Flaten TP, Odegard M (1988) Tea, aluminium and Alzheimer's disease. Food Chem Toxicol 26: 959-960.
42. Kim HK, Kim M, Kim S, Kim M, Chung JH (2004) Effects of Green Tea Polyphenol on Cognitive and Acetylcholinesterase Activities. Bioscience, Biotechnology, and Biochemistry 68: 1977-1979.
